# Interlobular and intralobular mammary stroma: Genotype may not reflect phenotype

**DOI:** 10.1186/1471-2121-9-46

**Published:** 2008-08-18

**Authors:** JM Fleming, EL Long, E Ginsburg, D Gerscovich, PS Meltzer, BK Vonderhaar

**Affiliations:** 1 Mammary Biology and Tumorigenesis Laboratory, Center for Cancer Research, National Cancer Institute, National Institutes of Health, Bethesda, MD 20892, USA; 2 Genetics Branch, National Cancer Institute, National Institutes of Health, Bethesda, MD 20892, USA

## Abstract

**Background:**

The normal growth and function of mammary epithelial cells depend on interactions with the supportive stroma. Alterations in this communication can lead to the progression or expansion of malignant growth. The human mammary gland contains two distinctive types of fibroblasts within the stroma. The epithelial cells are surrounded by loosely connected intralobular fibroblasts, which are subsequently surrounded by the more compacted interlobular fibroblasts. The different proximity of these fibroblasts to the epithelial cells suggests distinctive functions for these two subtypes. In this report, we compared the gene expression profiles between the two stromal subtypes.

**Methods:**

Fresh normal breast tissue was collected from reduction mammoplasty patients and immediately placed into embedding medium and frozen on dry ice. Tissue sections were subjected to laser capture microscopy to isolate the interlobular from the intralobular fibroblasts. RNA was prepared and subjected to microarray analysis using the Affymetrix Human Genome U133 GeneChip^®^. Data was analyzed using the Affy and Limma packages available from Bioconductor. Findings from the microarray analysis were validated by RT-PCR and immunohistochemistry.

**Results:**

No statistically significant difference was detected between the gene expression profiles of the interlobular and intralobular fibroblasts by microarray analysis and RT-PCR. However, for some of the genes tested, the protein expression patterns between the two subtypes of fibroblasts were significantly different.

**Conclusion:**

This study is the first to report the gene expression profiles of the two distinct fibroblast populations within the human mammary gland. While there was no significant difference in the gene expression profiles between the groups, there was an obvious difference in the expression pattern of several proteins tested. This report also highlights the importance of studying gene regulation at both the transcriptional and post-translational level.

## Background

Breast cancer is the most commonly diagnosed cancer, and is the second leading cause of cancer mortality in women in the U.S. [1]. Metastasis of the tumor is the primary cause of morbidity and mortality. In late-stage breast cancer, tumor metastasis can be found in several tissues, including bone, lung, lymph node, and liver [2]. Because metastasis is a major challenge in cancer management, a better understanding of the metastatic progression is required. Tumor progression and metastasis are both regulated by the surrounding microenvironment, i.e. the local stroma. Therefore, studies targeted towards understanding the function of normal breast stroma will facilitate the development of methods for preventing breast cancer metastasis.

Normal growth, function, and homeostasis of breast epithelial cells depend on intricate interactions between the numerous stromal cells within the mammary gland. The stromal cells are composed of a diverse assortment of cell types including the vasculature, adipocytes, resident immune cells, and fibroblasts. These cells secrete multiple cellular products, such as growth factors and extracellular matrix components, which have profound effects the behavior of the breast epithelial cells. Alterations in the regular communications between these cells can lead to the progression or expansion of malignant growth.

It is now well documented that stromal cells have a striking effect on the behavior of mammary epithelial cells in culture [3-7] as well as on the formation, growth, and metastasis of epithelial-derived tumors *in vivo *[8-11]. Both *in vitro *and *in vivo *studies have shown that epithelial cell contact with tumor-derived or normal fibroblasts can either promote or inhibit tumorigenic cell growth, respectively [11,12]. In agreement with these reports, one study using microarray analyses demonstrated that the gene expression profiles of cancer-derived fibroblasts had a distinctive gene expression pattern that differentiated them from normal breast stroma [13]. Furthermore, breast cancer stroma differs morphologically from the stroma found in normal breast tissue. For example, in ductal carcinomas *in situ *(DCIS), and most invasive breast carcinomas, the stroma exhibits enhanced accumulation of fibroblasts and a modified collagenized extracellular matrix compared to its normal counterpart [3,14-19]. Understanding the mechanisms of the interactions between cancerous or normal epithelial cells and the stroma might lead to novel methods for cancer therapies that target the function of the resident stromal cells.

Most models of breast cancer development are studied using mouse *in vivo *models. However, the stroma within the human mammary gland is fundamentally different from that in the mouse [20]. These differences make it difficult to ascertain the tumor/stromal interactions that would occur in the human breast when epithelial cells are implanted into the mouse mammary fat pad. Compared to the human breast, the mouse mammary gland contains large depots of adipose laced with small amounts of interspersed connective tissue. The functional lobular units of the mouse gland are embedded within the fat pad, and have a considerable amount of space between the minimally branched ducts. In contrast, the functional lobular units of the human mammary gland are surrounded by loose intralobular connective tissue, consisting primarily of fibroblasts. This intralobular stroma is subsequently surrounded by a more compact interlobular stroma, which detaches the lobules and intralobular stroma from any substantial direct contact with the adipose tissue [21]. Stemming from the observations that these stroma subtypes differ in their physical location in relation to the functional epithelial lobules, and that epithelial/stromal interactions can promote or inhibit tumorigenesis, we investigated the differences between the two distinct stromas.

## Methods

### Sample Collection

This study was performed in accordance with the guidelines of the National Cancer Institute Review Board, protocol 02-C-0144. All patients provided written informed consent. Fresh human mammary tissue was collected from four (two Caucasian, one African-American, and one Hispanic) female, pre-menopausal, reduction mammoplasty patients, ages ranging from 18 to 40 years old. The tissue was embedded in Tissue-Tek O.C.T. embedding medium (Sankura Finetek Inc., Torrance CA) and frozen on dry ice immediately after surgery. Eight – 10 micron sections of tissue were cut using a Leica 2800 Frigocut-E cryostat (Bannockburn, IL). Every tenth section was subjected to hematoxylin and eosin staining. For each patient sample, sections with distinctive intralobular and interlobular regions were selected for laser capture and microarray analysis.

### Laser capture microdissection and microarrays

Laser capture microdissection (LCM) and microarrays were performed by Cogenics, Inc. (Morrisville, NC). Briefly, selected intralobular and interlobular stroma sections of frozen tissue were subjected to an AutoPix™ automated LCM system from Arcturus, using static image settings. RNA was isolated from each specimen, pooled, and then evaluated by spectrophotometry and by using an Agilent Bioanalyzer before proceeding to sample amplification. For each sample, 50 ng of total RNA was amplified using Affymetrix Two-Cycle Target Labeling kit (Santa Clara, CA). Ten micrograms of biotinylated cRNA spiked with bioB, bioC, bioD, and cre as a control was hybridized to the Affymetrix Human Genome U133 GeneChip^® ^for 16 h at 45°C. Following hybridization, arrays were washed and stained with Affymetrix GeneChip Fluidics Station. Stained arrays were scanned with an Affymetrix GeneChip Scanner 3000. Quality check and preliminary data analysis were carried out using Affymetrix GeneChip Operating Software and Quality Reporter.

### Microarray analysis

Microarray data were analyzed using the Affy package available at the Bioconductor website . The raw data were first background-corrected by the Robust Multichip Average (RMA) method [22] and then normalized by an invariant set method. Unsupervised hierarchical clustering analysis was performed on 1,115 most variable genes. The difference of gene expression between the inter- and intra-stromal samples was analyzed by the Limma package available at the Bioconductor website. P-values obtained from the multiple comparison tests were corrected by false discovery rates. The microarray data has been deposited in the public repository, Gene Expression Omnibus, accession number GSE12306.

### Immunohistochemistry

All reagents were obtained from Sigma (St. Louis, MO) unless otherwise indicated. Ten-micron-thick sections of frozen tissue were fixed in 1:1 methanol:acetone for 10 min and washed in 1× phosphate buffered saline, pH 7.4 (PBS). Endogenous peroxidase activity was blocked by a 10 min incubation in 3% hydrogen peroxide followed by a 10 min wash in 1× PBS. Immunostaining was carried out using the Vectastain ABC kit (Vector, Burlingame, CA) according to the manufacturer's instruction. Color was developed with diaminobenzidine peroxidase substrate kit (Vector), and sections were counterstained with hematoxylin. Antibodies were obtained from the following sources: Met, Cell Signaling Technologies (Boston, MA); SOS2, Santa Cruz Biotechnology (Santa Cruz, CA); Tenascin-C, Invitrogen (Gaithersburg, MD); CD44, BD Biosciences (San Jose, CA); CD13, Novocastra (Visions Biosystems Bannockburn IL); CD26, Abcam Inc. (Cambridge, MA). Collagen staining with Sirius red was performed as previously described [23]. A positive and negative control was included in each experiment to validate the specificity of each antibody. A breast tissue sample that had been previously determined to express high levels of the protein of interest was used as a positive control. A serial section of each sample that received all staining steps, with the exception of the primary antibody, was used as a negative control.

### Reverse Transcription and PCR

Reverse transcription (RT) reactions were performed with 0.5 μg of total RNA isolated from LCM using Moloney murine leukemia virus reverse transcriptase (Invitrogen) primed with oligo-dT and random hexamers in a final volume of 25 μL. PCR was performed on 2.5 μL RT product using PCR Master Mix (Roche, Indianapolis, IN) with 0.2–0.4 μM of each primer. Primer sequences can be found in Table 1. Conditions for each PCR reaction were as follows: 94°C for 3 min for one cycle, followed by 94°C for 1 min, 60°C for 1 min, 72°C for 2 min, with a final extension at 72°C for 10 min. For a given experiment, PCR was performed using a predetermined number of cycles that spanned the linear range for the samples tested (20–30 cycles). RT-PCR products were resolved by agarose gel electrophoresis, visualized with ethidium bromide, quantified using NIH Image, and normalized to the respective level of GAPDH mRNA. Semi-quantitative RT-PCR analyses were conducted on a minimum of three patient samples. Appropriate negative controls were included for each RT-PCR.

**Table 1:** Primer sequences

**Primer**	**Primer sequences (listed 5' – 3')**
GAPDH	Forward CATGTGGGCCATGAGGTCCACCAC Reverse TGAAGGTCGGTGTGAACGGATTTGGC
c-Met	Forward ACCTGCTGAAATTGAACAGCGAGC Reverse ACACTTCGGGCACTTACAAGCCTA
SOS2	Forward TAGAGAAAGGCGAGCAGCCAATCA Reverse AGGGTGAGATTTGTGGTATGGCGA
CD44	Forward GCCTGGCGCAGATCGATTTGAATA Reverse CCCTGTGTTGTTTGCTGCACAGAT
Tenascin-C	Forward AGATGTCACAGACACCACTGCCTT Reverse TGTGGCTTGTTGGCTCTTTGGAAC
CD13	Forward TCCACACCTTTGCCTACCAGAACA Reverse TGCCTGATGTGCTGAAGAGATCGT
CD26	Forward TGGAGGCATTCCTACACAGCTTCA Reverse ACAGCTCCTGCCTTTGGATATGGA

## Results and discussion

**Figure 1:**
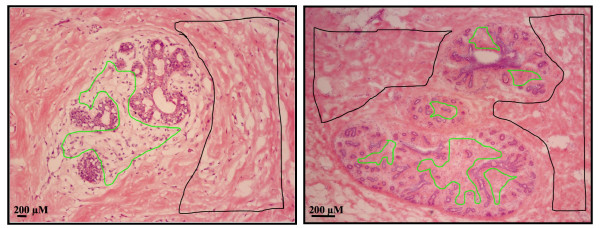
**Identification of intralobular and interlobular stroma in normal human breast tissue**. Hemotoxylin and eosin staining of 8–10 micron sections of normal mammary tissue. The intralobular stroma isolated for laser capture microscopy is outlined in green while the interlobular stroma is outlined in black. Scale bar = 200 μM.

**Figure 2:**
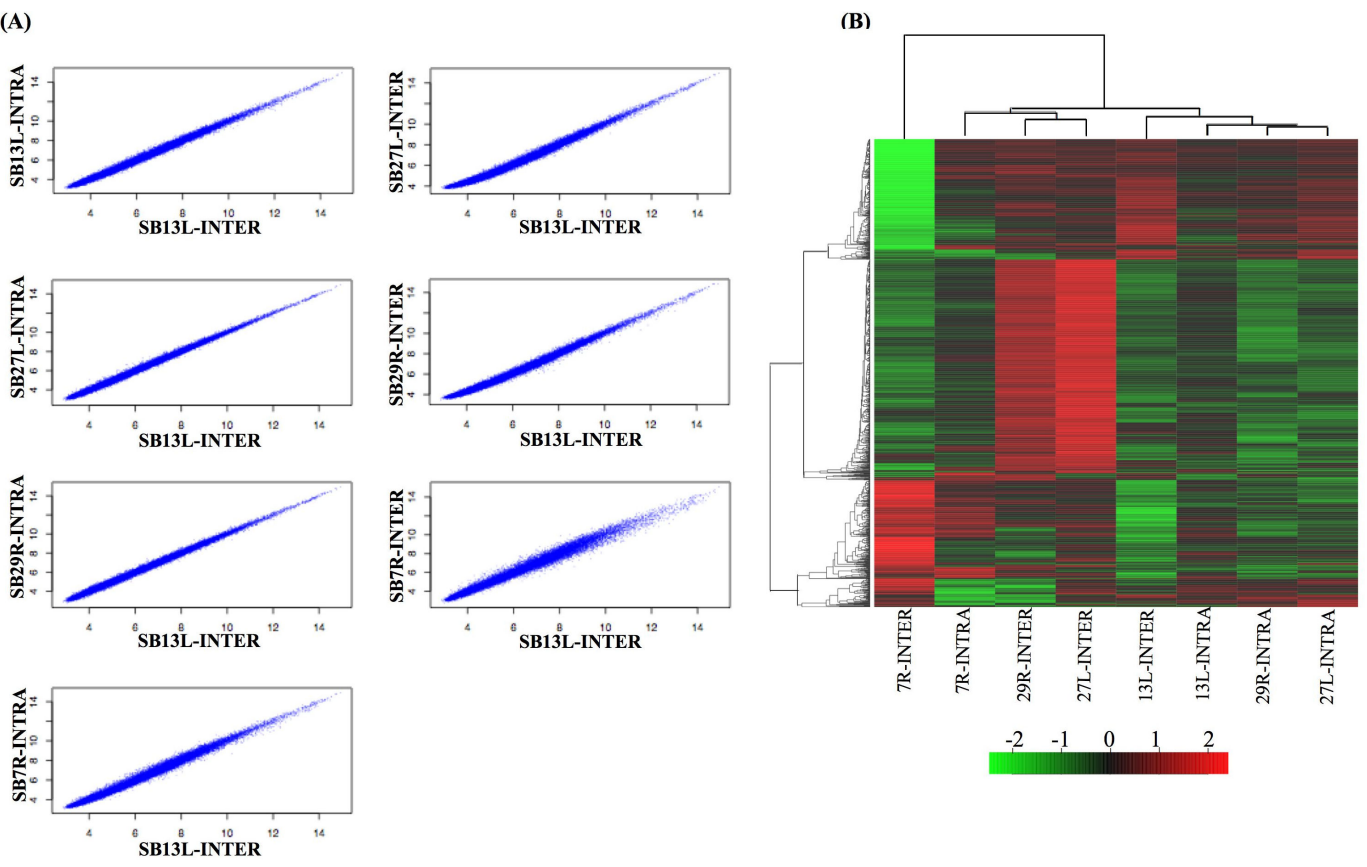
**Scattered plots of normalized data and unsupervised hierarchical clustering of the samples and genes**. A. Raw intensity data was background-corrected and normalized as described in Materials and Methods. The normalized data from seven samples were plotted against one sample (SB13L-Intra). B. 1,115 most variable genes were used for hierarchical clustering among samples. The gene expression values were scaled by row and shown in the heat map.

**Figure 3:**
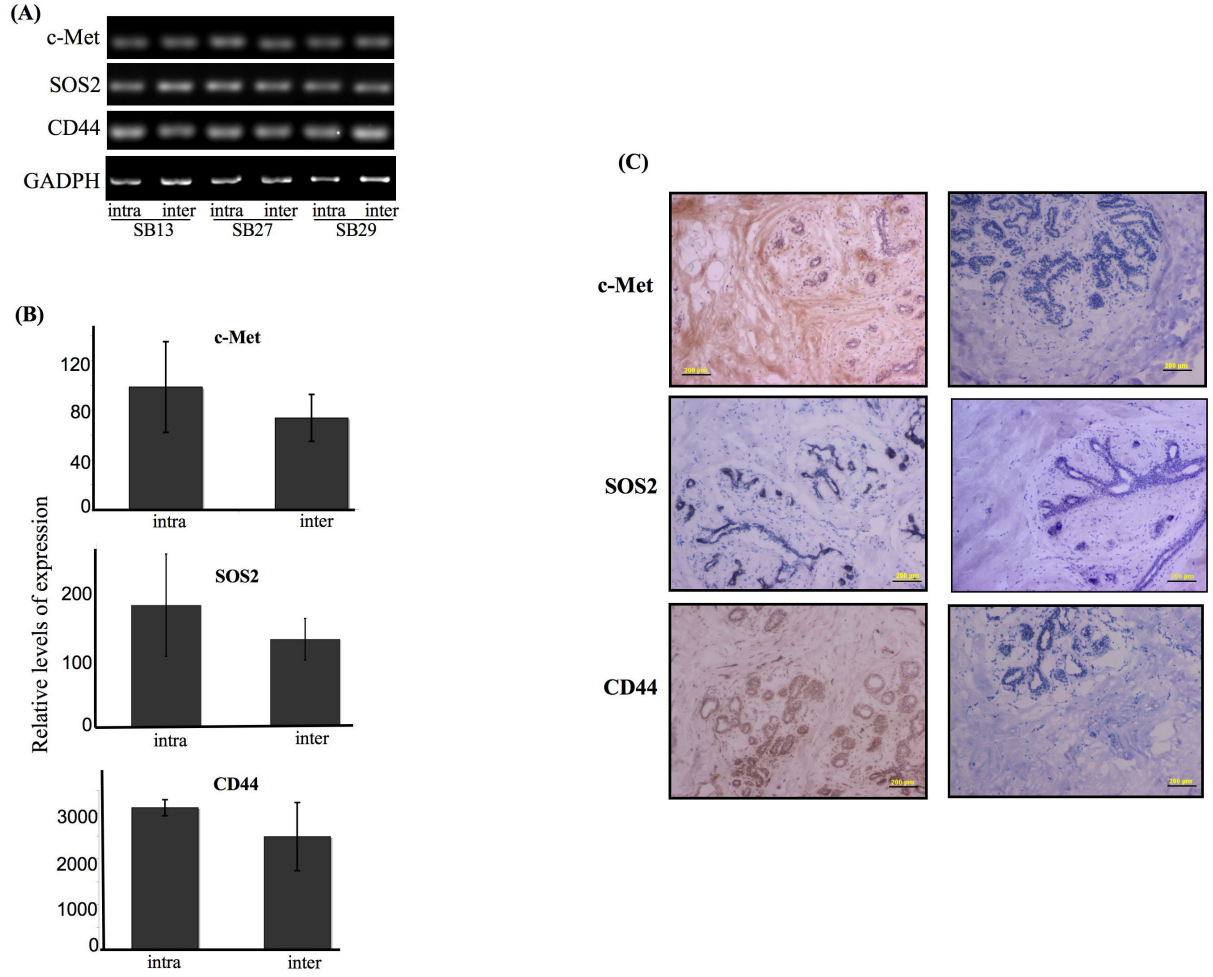
**c-Met, SOS2, and CD44 expression levels in intralobular and interlobular normal human breast stroma**. A. RT-PCR analysis of the indicated genes expression. B. Mean ± SD gained by densitometric examination of RT-PCR product from three independent samples. C. Tissues were subjected to immunohistochemical analysis with the specific antibody indicated (left panels) or corresponding negative controls (right panels). Scale bar = 200 μM.

Microarray analysis was employed to identify genes that were differentially regulated between the intralobular and interlobular stromal subtypes. Normal human mammary tissue was obtained from healthy, pre-menopausal, reduction mammoplasty patients with no incidence of neoplasia. For each sample collected, tissue sections with distinctive intralobular and interlobular regions were selected for laser capture and microarray analysis (Fig. 1). RNA was extracted from the samples, checked for quality, and then hybridized with the Affymetrix Human Genome133A GeneChips, containing over 22,000 oligonucleotide probes. Surprisingly, no significant difference in gene expression was found between the two stroma subtypes. The microarray data demonstrated a wide range of expression values and a 45-degree straight line in each pair of samples, indicating the microarray assay and the normalization procedure were valid. Despite the small sample number, the scatter plots showed limited spread of the off-diagonal lines, suggesting that any differential expression between samples is subtle, and not significant (Fig. 2A). A heatmap with dendrograms was also generated from the data (Fig. 2B). At first glance, genes from sample numbers 13L, 27L, and 29R appeared to separate as different clusters with respect to interlobular and intralobular samples. However, further in-depth analysis using hierarchical clustering of the samples, based on the 1,115 most variable genes, did not reveal a distinct expression pattern between the intralobular and interlobular stromal tissues, further indicating there was no significant difference in terms of gene expression at the transcriptional level. Six of the genes with the largest difference in expression levels between intralobular in interlobular stroma are listed in Table 2, along with the fold-change and p-value. The lowest p-value was found to be 0.4726, which is far from statistically significant. In order to validate the microarray data, RT-PCR was performed on three of the top six genes listed in Table 2. The RT-PCR products from three patient samples as well as the quantitation of the products are shown in Figure (3A&3B). The expression levels of c-Met, SOS2, and CD44 reflect the findings of the microarray data; there was no significant difference between the intralobular and interlobular stroma.

**Table 2:** Top six genes with the highest p-value

**Gene name**	**Accession number**	**Log fold change**	**P-value**
C-Met	AA005141	-0.4891	0.4726
SOS2	AI276593	-0.4345	0.7845
CPT1A	BC000185	-0.4465	0.7845
PDLIM7	AW206786	-0.3722	0.7845
TSC22D2	AF201292	-0.4455	0.7845
CD44	AW851559	-0.4704	0.7845

### Examples of protein levels reflecting the gene expression levels between intralobular and interlobular stroma

While the microarray and supportive RT-PCR analysis revealed no significant difference between gene expression levels, previous reports have documented a distinctive immunohistochemical difference between intralobular and interlobular stroma [23-27]. Therefore, we investigated whether we could observe a similar phenomenon using the same patients tissue samples utilized in the microarray analysis. We first investigated the protein expression of c-Met, the gene with the smallest p-value (0.4726). Fixed preparations of human mammary tissue from the four patients used in the microarray analysis, as well as additional samples, were immunoassayed using a specific antibody for c-Met. As shown in Figure 3C, all of the stroma uniformly stained positive for c-Met protein expression, with no detectable difference between the two stroma subtypes. The c-Met gene encodes the tyrosine kinase receptor for the hepatocyte growth factor/scatter factor (HGF/SF). c-Met/HGF signaling is required for mammalian embryogenesis and is important in cell migration, morphogenic differentiation, cell growth and angiogenesis. In normal breast tissue, c-Met was reported to be associated with ductal cells, and involved in ductal branching [28]. Additionally, the overexpression of c-Met has been shown to contribute to the development and progression of different human malignancies including lung, prostate, colorectal, gastric, and breast cancer [29]. Recently, it was reported that c-Met protein is overexpressed in inflammatory breast cancer compared to non-inflammatory breast cancer, and that an imbalance of c-Met protein expression between tumor and surrounding normal tissue is associated with an aggressive DCIS phenotype [30,31]. In the present study, c-Met protein was easily detectable and uniformly distributed throughout the normal breast.

We next investigated the protein expression pattern of SOS2 (Son of Sevenless), the gene with the second lowest p-value (0.7845). SOS2 is a Ras-specific nucleotide-exchange factor that is involved in the receptor tyrosine kinase-Ras-ERK cascade [32]. This cascade has been implicated in the control of diverse biological processes including cell proliferation, differentiation, and survival. All tissues immunoassayed for SOS2 showed sparse, weak, staining in the stroma with no detectable difference in the staining pattern between the stroma subtypes (Fig. 3C). The only significant positive staining was found in the luminal epithelium of each sample.

CD44, a protein which has recently gained much attention in breast cancer [33-37], is a ubiquitously expressed, multifunctional cell surface adhesion molecule involved in cell-cell and cell-matrix interactions, cell trafficking, and transmission of numerous growth signals [38]. The primary ligand for CD44 is hyaluronic acid, which is an important component of the extracellular matrix. However, other CD44 ligands include collagen, fibronectin, laminin, and chondroitin sulfate. Stromal hyaluronic acid levels are a strong, independent, negative predictor for patient survival in breast cancer [39,40]. Additionally, many cancer cells overexpress CD44 or express CD44 variants [41]. Mouse models of breast cancer tumorigenicity have suggested that CD44 expression is a cell surface marker that differentiates tumor initiating from non-tumorigenic breast cancer cells in immuno-compromised mice [42]. Furthermore, injection of reagents interfering with CD44-ligand interaction, such as CD44-specific antibodies, has been shown to inhibit local tumor growth and metastatic spread in mouse models of human cancer [38]. These findings suggest that CD44 may confer a growth advantage on some neoplastic cells and, therefore, could be used as a target for cancer therapy. In the present study, CD44 was one of the top genes with differential regulation between intralobular and interlobular stroma, although the p-value was 0.7845 and not significant. Immunohistochemical analysis reflected the microarray and RT-PCR results. There was uniform staining of the stroma, and the highest immuno-reactivity for CD44 was found in the epithelial cells (Fig. 3C).

### Examples of proteins differentially regulated between the intralobular and interlobular stroma, with no significant change in gene expression

**Figure 4:**
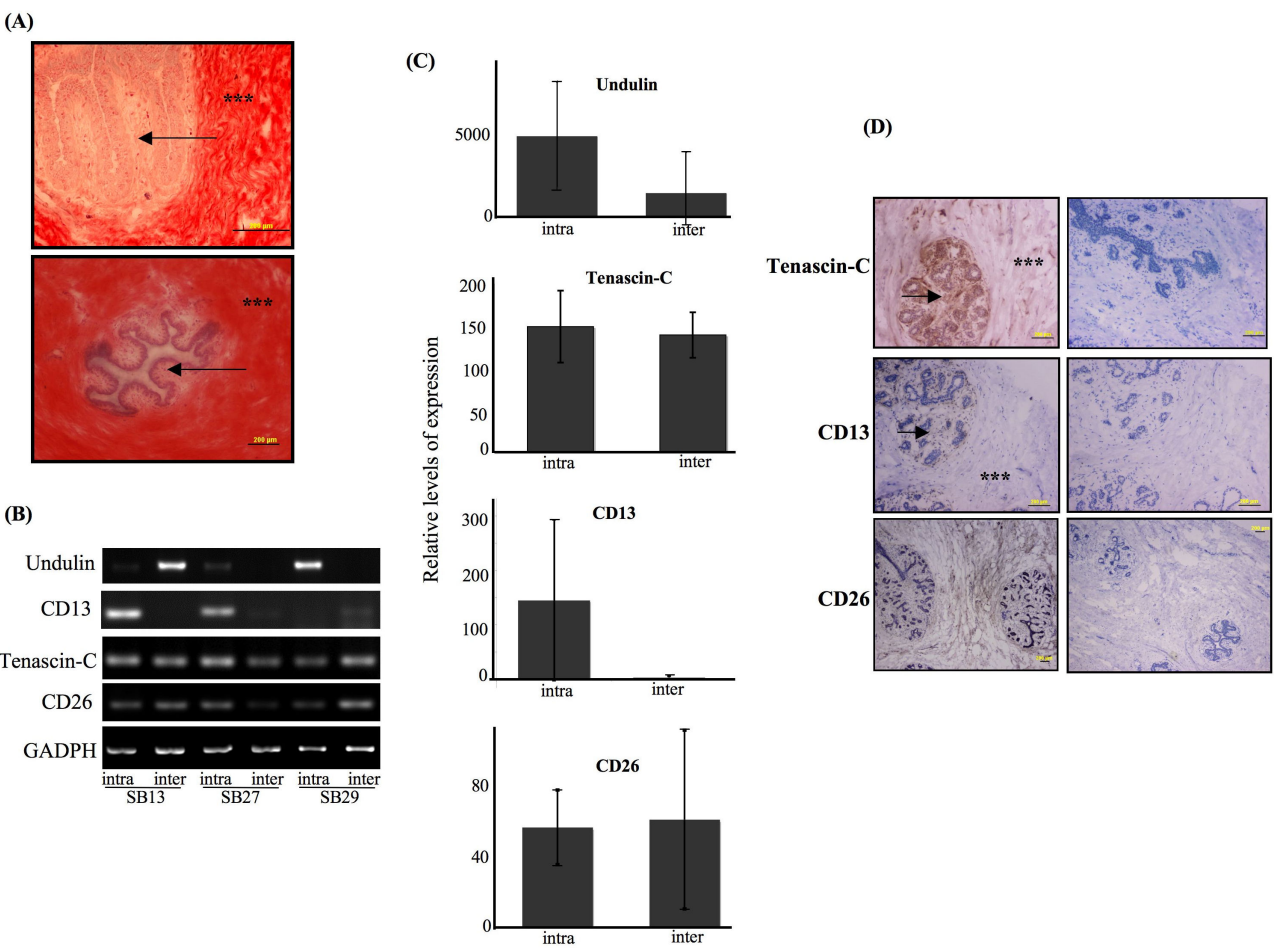
**Localization and expression levels of collagen fibrils, CD13, Tenascin-C, and CD26 in intralobular and interlobular normal human breast stroma**. A. Tissues were stained with Sirius Red alone (top panel) or with Fast Green counterstain (bottom panel). B. RT-PCR analysis of the indicated genes expression. C. Mean ± SD gained by densitometric examination of RT-PCR product from three independent samples. D. Tissues were subjected to immunohistochemical analysis with the specific antibody indicated (left panels) or corresponding negative controls (right panels). Note large quantities of intensely stained interlobular stroma (asterisks) compared to the paler-staining intralobular stroma (arrow). Scale bar = 200 μM.

As previously stated, several reports illustrate a difference in protein expression between the intralobular and interlobular stroma. Atherton *et al*. (1998) reported that immuno-localization of type XIV collagen/undulin in the human mammary gland revealed greater deposition in the interlobular stroma than in the intralobular stroma [25]. Fibroblasts isolated from the interlobular stroma synthesized 3- to 5-fold more type XIV collagen/undulin than intralobular fibroblasts, but synthesized type I and type IV collagens in similar amounts. The authors suggest this protein is a way to separate the two types of distinct stroma for analysis. Collagen fibers have also been reported to be more abundant and densely packed throughout interlobular stroma compared to intralobular stroma in the bovine mammary gland [23]. Thus, we examined the collagen fiber deposition in the tissue samples from the patients used in the microarray data. Using Sirius Red, a pan stain for collagen fibers, there was a clear visible difference in the deposition of collagen fibers between the two types of stroma (Fig. 4A). In our microarray data, the fold change and p-value for type XIV collagen/undulin were -0.199 and 0.785, respectively. Undulin had the best p-value compared to all other types of collagen, but again, no values for any of the collagen genes were significant. RT-PCR analysis of the patients samples used in the microarray revealed an inconsistent expression of undulin between samples, resulting in no significant change between intralobular and interlobular expression (Fig. 4B&4C).

Our current data illustrated that the interlobular stroma has increased stromal collagen compared to the intralobular stromal. Mammographically dense breast tissue is one of the greatest risk factors for developing breast carcinoma, and regions of high breast density are associated with increased stromal collagen [43-45]. A recent report investigating the effects of collagen density on mammary tumor formation and progression utilized a bi-transgenic tumor model with increased stromal collagen in mouse mammary tissue [46]. This increased stromal collagen significantly increased tumor formation and resulted in a significantly more invasive phenotype, with increased lung metastasis. This study provided the first data causally linking increased stromal collagen to mammary tumor formation and metastasis, and demonstrated that fundamental differences arise and persist in epithelial tumor cells that progressed within collagen-dense microenvironments. It could be hypothesized that a change in the protein expression of the intralobular stroma to mimic the collagen expression of the interlobular stroma would enhance breast cancer progression. Studying the mechanisms, which lead to the differential levels in collagen deposition between these two stromal subtypes, could facilitate in understanding the physiology of breast density and the resultant influences on mammary epithelial cell function.

Tenascin-C has also been reported to be expressed in the intralobular stroma as well as in the basement and sub-basement membrane zone of normal breast tissue [47]. Tenascin-C is a member of the tenascin family of modular and multifunctional extracellular matrix glycoproteins. These molecules are expressed in the adult during normal processes such as wound healing and tissue involution, and in pathological states including vascular disease, tumorigenesis, and metastasis [48]. In the present study, there was substantially more immuno-staining in the intralobular stroma compared to the interlobular stroma (Fig. 4D). Both the sub-basement membrane as well as the stroma had higher immuno-reactivity compared to the interlobular stroma. The microarray data reported a -0.119 fold change and a p-value of 0.852 for tenascin-C. RT-PCR was also performed on the same patient samples, and similar to the microarray data, showed no significant difference in tenascin-C expression (Fig. 4B&4C). Tenascin-C has been reported to be overexpressed in the extracellular matrix of the stroma in many solid tumors, including breast tumors [49,50]. Additionally, expression of tenascin-C in DCIS has been demonstrated to predict invasion, and high expression has been related to poor prognosis, as well as local and distant reoccurrence in breast cancer patients. [51-55]. Interestingly, both the distribution and quantity of tenascin-C changes in the breast during the menstrual cycle [47], which may explain the variations in tenascin staining in normal tissue, as well as hormone-dependent and independent tumors. Although intralobular stroma was reported to undergo cyclic changes during the menstrual cycle [56], there was no measurable difference in protein levels of the estrogen and progesterone receptor status within the two types of stroma (*data not shown*). Similar to the results seen with the collagen deposition, this is another example of gene expression levels that do not reflect the abundance of the protein between the two types of stroma.

Atherton *et al*. (1994) have reported a unique regulation of ectoenzymes between the intralobular and interlobular stroma [26]. In normal breast tissue, aminopeptidase N (CD13) was reported to be uniformly expressed in all stroma, while dipeptidyl peptidase IV (CD26) was absent in the intralobular stroma, but present in the interlobular stroma. The two subpopulations of stromal cells were isolated by enzymatic digestion and cell culture, and then analyzed via flow cytometry and immunohistochemistry. Interestingly, after several passages on tissue plastic culture dishes, the intralobular stroma lost their expression of CD26 and became phenotypically similar to the interlobular fibroblasts. This suggests that growth on tissue culture plastic causes a reversion of the stroma subpopulations to one phenotype. We subjected the patient samples from the microarray data to both immunohistochemistry and RT-PCR for both CD13 and CD26. In contrast to Atherton's report, immunohistochemical analysis of CD13 illustrated a predominately intralobular stroma staining in all patient samples tested (Fig. 4D). However, similar to the microarray data, RT-PCR analysis showed inconsistent expression levels between patients (Fig 4B&4C). Of the four patients used in the microarray analysis, two samples had higher CD13 expression in the intralobular stroma, while the other two had higher expression in the interlobular stroma. A larger sample size is necessary to determine whether the RT-PCR results were significant. Additionally, the intensity of the immunoreactivity may be attributed to the density of the stromal cells between the stroma subtypes, and the overall stromal density of each patient may influence the immunohistochemical analysis and a larger sample size is required for absolute conclusion.

As with CD13, CD26 demonstrated inconsistent staining between samples, without specific staining to the intralobular or interlobular stroma (Fig. 4D). In some patient samples, CD26 demonstrated a slightly greater deposition in interlobular stroma than intralobular stroma, while in other samples CD26 was ubiquitously expressed throughout all stroma. The RT-PCR results reflected the inconsistency of CD26 protein expression, and similar to the microarray data, quantitation of the samples resulted in no significant difference in expression (Fig. 4B&4C).

## Conclusion

Recently it was reported that the gene expression signatures of cancer-adjacent and breast reduction-normal tissues were essentially homogeneous and not distinguishable [57]. The stroma used in this microarray study was exclusively interlobular stroma, and specifically excluded any intralobular stroma. The authors state this was the most complete study to date of gene expression in normal breast tissue, and that normal tissue adjacent to breast carcinomas has not undergone significant gene expression changes. However, the present study highlights the importance of post-transcriptional or post-translational regulation of proteins. Since surgery is a common procedure performed on tissue with potential for tumor progression, the alterations in the adjacent stroma could have important clinical implications. This study emphasizes the importance of using techniques other than gene expression levels to investigate protein regulation within the stroma.

A recent report utilizing two-dimensional gel electrophoresis supports the present study and shows that carcinoma-associated fibroblasts, tumor-adjacent fibroblasts (cells 2 cm away from the tumor margin), and normal breast fibroblasts have different proteome profiles, with many different proteins differentially expressed among these cells [58]. Interestingly, the carcinoma-associated fibroblasts and tumor-adjacent fibroblasts expressed high levels of the cancer marker survivin and consequently exhibited high resistance to the chemotherapeutic agent cisplatin and UV light. Furthermore, the tumor-adjacent fibroblasts, although histologically normal and not in contact with the tumor cells, contained genetic changes that were distinct from the normal fibroblasts and the carcinoma-associated fibroblasts. It was hypothesized that the carcinoma-associated fibroblasts, as well as their corresponding tumor-adjacent fibroblasts, acquired tumor-like changes that are necessary for tumor growth. The authors further speculated that certain genes are up-regulated early during carcinogenesis and have a promoting role during cancer development. It would be of interest to investigate, based on the data obtained from the present study, whether the carcinoma-associated fibroblasts and tumor-adjacent fibroblasts arise from the intralobular or the interlobular stroma, and what effects tumorigenic changes in either subtype have on each other and the progression of the cancer.

The failure to grow normal or premalignant human mammary epithelial cells *in vivo *had previously hindered any possibility of a model for human breast cancer progression using human cells. Recently, Kuperwasser *et al*. [12] successfully developed a dynamic *in vivo *model which recapitulates human breast epithelial morphogenesis. In this model, human mammary fibroblasts are injected into the gland and allowed to grown into the gland and "humanize" the mouse fat pad, prior to injection of the epithelial cells. This model demonstrated that stroma promoted the normal or premalignant to malignant growth of the epithelial cells, depending on the type of fibroblasts used. It may be informative to observe the differences in the normal outgrowth or tumorigenesis of epithelial cells when either intralobular or interlobular fibroblasts are chosen to humanize the gland. Furthermore, future studies isolating the differences between these two stromal subtypes may bring further insight into the tumor/stroma environment as well as normal mammary development.

## Abbreviations

LCM: Laser capture microscopy; RT-PCR: reverse transcription polymerase chain reaction; HGF/SF: hepatocyte growth factor/scatter factor; SOS2: Son of Sevenless; CD13: aminopeptidase N; CD26: dipeptidyl peptidase IV

## Competing interests

The authors declare that they have no competing interests.

## Authors' contributions

JF performed immunohistochemistry, RT-PCR, and drafted the manuscript. LL performed the analysis on the microarray data. EG participated in the design of the study, collection and distribution of patient samples, and helped edit the manuscript. DG prepared the samples for laser capture microscopy, PM and BKV designed the experiments and helped interpret the results. All authors read and approved the final manuscript.
